# Culture and End of Life Care: A Scoping Exercise in Seven European Countries

**DOI:** 10.1371/journal.pone.0034188

**Published:** 2012-04-03

**Authors:** Marjolein Gysels, Natalie Evans, Arantza Meñaca, Erin Andrew, Franco Toscani, Sylvia Finetti, H. Roeline Pasman, Irene Higginson, Richard Harding, Robert Pool

**Affiliations:** 1 Barcelona Centre for International Health Research, Universitat de Barcelona, Barcelona, Spain; 2 Department of Palliative Care, Policy and Rehabilitation, King’s College London, London, United Kingdom; 3 Fondazione “Lino Maestroni”, Istituto di Ricerca in Medicina Palliativa, Cremona, Italy; 4 Centre for Social Science and Global Health, University of Amsterdam, Amsterdam, The Netherlands; 5 Department of Public and Occupational Health, Emgo Institute for Health and Care Research, Expertise Center for Palliative Care, VU University Medical Center, Amsterdam, The Netherlands; Universidad Europea de Madrid, Spain

## Abstract

**Aim:**

Culture is becoming increasingly important in relation to end of life (EoL) care in a context of globalization, migration and European integration. We explore and compare socio-cultural issues that shape EoL care in seven European countries and critically appraise the existing research evidence on cultural issues in EoL care generated in the different countries.

**Methods:**

We scoped the literature for Germany, Norway, Belgium, the Netherlands, Spain, Italy and Portugal, carrying out electronic searches in 16 international and country-specific databases and handsearches in 17 journals, bibliographies of relevant papers and webpages. We analysed the literature which was unearthed, in its entirety and by type (reviews, original studies, opinion pieces) and conducted quantitative analyses for each country and across countries. Qualitative techniques generated themes and sub-themes.

**Results:**

A total of 868 papers were reviewed. The following themes facilitated cross-country comparison: setting, caregivers, communication, medical EoL decisions, minority ethnic groups, and knowledge, attitudes and values of death and care. The frequencies of themes varied considerably between countries. Sub-themes reflected issues characteristic for specific countries (e.g. culture-specific disclosure in the southern European countries). The work from the seven European countries concentrates on cultural traditions and identities, and there was almost no evidence on ethnic minorities.

**Conclusion:**

This scoping review is the first comparative exploration of the cultural differences in the understanding of EoL care in these countries. The diverse body of evidence that was identified on socio-cultural issues in EoL care, reflects clearly distinguishable national cultures of EoL care, with differences in meaning, priorities, and expertise in each country. The diverse ways that EoL care is understood and practised forms a necessary part of what constitutes best evidence for the improvement of EoL care in the future.

## Introduction

Given the ageing of European populations, there will be a growing demand for end of life (EoL) care in the coming years [1], [2]. In a context of globalization, migration and European integration, culture is becoming increasingly important in relation to health care. It affects patients’ and professionals’ perceptions of health conditions and appropriate treatments, and it influences responses to illness, health care services and death [3], [4]. When patients, their families and health professionals face chronic or terminal illness, with limitations to cure and difficult decisions, differences in cultural norms and values become especially salient.

In order to improve care [5], [6], and ensure that palliative care can secure its share from national budgets and allocate these resources in a just way [7] there is a need for relevant evidence. The urgent calls for an evidence-base have already led to increasing research activity in clinical practice and service provision [7], [8], [9], [10]. The history of the hospice movement, originally developed in the UK, has been well described [11], [12] and the adoption of the hospice model in the rest of the Anglo-Saxon world is equally well documented [13]. More recently, initiatives have started to map developments in EoL care in Europe and the rest of the world with a focus on service-provision [14], [15]. Less is known about the socio-cultural context in which EoL care is developing. By including a section on culture and EoL care the Economist Intelligence Unit’s comparative report recognised its importance for EoL care [1]. However, no attempt to explore this area more systematically in an international context has been made so far. This paper addresses evidence on the role of culture in EoL care in Europe.

European countries are in different stages of developing palliative care provisions and services take various organisational forms within health systems [1]. This is a consequence of different cultural traditions and attitudes towards the EoL and related care [16]. The differences in services can lead to further diversification in the understanding of EoL care across Europe, which presents challenges for research and international collaborations to improve EoL care on a wider scale.

This paper reports on a scoping exercise of cultural issues in EoL care of seven European countries: Germany, Norway, Belgium, the Netherlands, Spain, Italy and Portugal. These are seven of the eight participating countries in the PRISMA project, in the context of which this work was undertaken [17]. The evidence of the eighth country, the UK, was published separately [18], [19], because it is so different from the other European countries and therefore not comparable with the same criteria. The focus of this scoping exercise of the European countries was two-fold. First, we aimed to explore and compare socio-cultural issues that shape EoL care in each of the countries. Second, we aimed to critically appraise the research evidence on cultural issues in EoL care produced in the different countries to throw light on its adequacy as a basis for the further development of EoL care.

## Methods

### Approach

A scoping exercise with the purpose of aggregating and interpreting the evidence on culture and EoL care in the seven targeted European countries. This type of review is suitable to map evidence in a broad topic area which has not been reviewed before. The review was exploratory and applied an iterative and inductive approach. Therefore it did not specify concepts in advance of the synthesis, but let the delineation of the phenomenon of culture in relation to EoL care emerge in the analysis of the literature. We started with an open and broad review question, which was refined by the search results and the findings in the studies retrieved.

### Search Strategy

A team of researchers undertook some pilot searches, separately for each country, to get an idea of the scope of the literature that informed about culture and EoL care for each country, and the suitability of the search terms. The following search terms were used for the pilot searches:

*Country name(s):*

(Germany OR German*)

(Norway OR Norwegian*)

(Belgium OR Belg*)

(Netherland* OR Dutch OR Holland)

(Spain OR Spanish*)

(Italy OR Italian*)

(Portugal OR Portug*)

**AND**

(palliative OR terminal OR “end of life” OR end-of-life OR death OR dying OR “continu* care” OR “advance directive*” OR hospice* OR “supportive care”)

**AND**

(cultur* OR intercultural OR cross-cultural OR transcultural OR qualitative OR ethnography OR anthropology OR interview* OR “focus group*”)

These were chosen with the aim of retrieving articles concerning EoL care where cultural and social factors were sufficiently relevant to be referred to in the title, abstract, topic or key words.

These initial pilot searches retrieved bodies of literature of varying sizes from each country and this was compared and discussed in a team meeting. For Belgium, Spain, Italy and Portugal, so few articles were retrieved that it was necessary to make additional, more general searches. The definitive searches were conducted in the following electronic databases:

Web of Knowledge all databases (Web of Science with conference Proceedings (1899–2012), BIOSIS Previews (1969–2012), Inspec (1969–2012), MEDLINE (1950–2012), Journal Citation Reports (2000–2012)); OVID (AMED (1985–2012); PsycINFO (1806 to 2012); and EMBASE (1980 to 2012)); Cancerlit (1975–2012); ASSIA (1987–2012); and, CINAHL (1982 to 2012).

#### Electronic search for Belgium, Spain, Italy and Portugal

Due to the small size of the body of literature retrieved by the database search of Belgian, Spanish, Italian, and Portuguese literature, more general searches unrestricted by the terms relating to culture, were carried out using the search terms:

(Belgium OR Belg*)

(Spain OR Spanish*)

(Italy OR Italian*)

(Portugal OR Portug*)

**AND**

(palliative OR terminal OR “end of life” OR end-of-life OR death OR dying OR “continu* care” OR “advance directive*” OR hospice* OR “supportive care”)

In addition, a number of country-specific databases were used when available, (see Table 1). The searches were updated to February 2012.

**Table 1:** Journals hand searched.

Country	Journals, conference indices, or websites subjected to hand searches
**Germany**	Omega Volume 1 Number 1 (1970) to Volume 58 Number 1 (2008); Mortality Volume 1 Issue 1 (1996) to Volume 13 Issue 4 (2008); Medical Anthropology Volume 21 (2002) to Volume 28 (2009).
**Norway**	Omega Volume 1 Number 1 (1970) to Volume 58 Number 1 (2008); Mortality Volume 1 Issue 1 (1996) to Volume 13 Issue 4 (2008); Scandinavian Journal of Caring Sciences Volume 15 Issue 1 (2001) to Volume 23 Issue 4 (2009); Medical Anthropology Volume 21 (2002) to Volume 28 (2009).
**Belgium**	Revue Médicale de Bruxelles, Ethical Perspectives: Issue 2–3/2002; Tijdschrift voor Geneeskunde; and Acta Hospitalia.
**Netherlands**	Non accessible (due to language limitations).
**Spain**	Spanish medical anthropology bibliography (available in Perdiguero and Comelles (2000)); Spanish National Conferences of Anthropology; The REDAM conferences; and The Medical Anthropology at Home Conferences; Spanish Society of Palliative Care website (SECPAL); Basque Society of Palliative Care website (SOVPAL); Spanish Association Against Cancer website (AECC)
**Italy**	Not included.
**Portugal**	Associação Portuguesa de Cuidados Paliativos website; História dos Cuidados Paliativos em Portugal website; ONCO.news (Associação de Enfermagem Oncológica Portuguesa - AEOP).

#### Other searches

Hand searches were carried out in reference lists of retrieved articles and cited reference searches were conducted. Archives of key journals were searched, in order to find relevant articles that had been missed in the initial database search. Journals were selected for hand searches if they contained a high frequency of relevant articles, identified in the electronic, hand and cited references searches (see Table 2).

**Table 2:** Additional databases by country.

Country	Additional databases
**Germany**	Non accessible (due to language limitations).
**Norway**	Non accessible (due to language limitations).
**Belgium**	CSA Illumina
**Netherlands**	CSA Illumina
**Spain**	IME (medicine); ISOC (social sciences); CUIDEN (nursing) and ENFISPO (nursing); Pubmed and Current Contents.
**Italy**	Non accessible
**Portugal**	IME (medicine); ISOC (social sciences); CUIDEN (nursing).

Publications written by authors of the articles deemed relevant were searched via authors’ web pages (if available) and the Web of Knowledge ‘author search’ facility.

Searches were also conducted in a number of Spanish and Portuguese web pages dedicated to palliative and cancer care and these were categorised as ‘hand searches’ as the web pages had no search facility and the literature available via the web pages was explored manually. For Spain, the full medical anthropology bibliography [20], and medical anthropology conferences were also hand-searched.

Grey literature –documents that are disseminated outside standard publication channels such as scientific journals but which have a definite influence on scientific output (for example policy reports or conference proceedings)– was obtained from experts identified from the expert network on culture and EoL care that was created concurrently to the scoping as part of the PRISMA project.

### Screening and Data Extraction

All documents were considered for relevance based on titles and abstracts. When the information was not sufficient to decide on inclusion or exclusion, the full text was evaluated. Because of the dearth of evidence in this area and the exploratory nature of the scoping of this literature, the team first became familiar with the literature from each country and then discussed inclusion and exclusion criteria in a team meeting. This was then used as a guide for deciding about their relevance to the review question. We included reviews and original research studies that informed about socio-cultural issues in EoL care. We excluded studies on clinical tools, pain and symptom management, pharmaceuticals, donation and transplants, neonatal EoL issues, legal issues and psychology. However if any of these studies contained relevant elements they were read fully and included. The electronic searches were restricted to the English language. Handsearches in the national languages were conducted for Spain, Portugal, Italy, Germany, Belgium and the Netherlands. To optimise comparability between countries we restricted the review to papers that were produced after the arrival of the ideas of the palliative care movement.

Data extraction was conducted for all studies, which inventorised study details, participants, methodology, and main findings.

### Synthesis

The initial stage was mainly oriented towards examining the extent, range and nature of research activity, although this already implies interpretation and formed a part of the synthesis of the findings. A mapping of reported practices and concepts was carried out regarding cultural issues in EoL care for each country through techniques of identification, listing, tabulating and counting individual studies. The overviews and opinion pieces were from this point not systematically analysed, although they were read as background literature and informed the further stages of synthesis. Themes across multiple studies were developed inductively through constant comparison of findings. Each team member developed a country-specific narrative synthesis.

Second, the country-specific syntheses were exchanged among the members of the team and read. Each developed a framework of themes that was capable of integrating all the themes used in the syntheses of the other countries, retaining the capacity to still compare findings across countries in a meaningful way. These frameworks were then discussed in team meetings and a common framework was agreed upon, consisting of five themes: setting, caregivers, communication, medical EoL decisions, minority ethnic groups, and knowledge, attitudes and values of death and care. The narrative syntheses for each country were then rewritten according to these cross-cutting themes. This assisted the comparison of practices, and ideas related to developments in EoL care across the countries and the identification of similarities or differences in approaches.

A third step focused on the body of literature itself, as a source of information on how the evidence approaches and thereby constructs the issues it addresses. Then, by examining the number and type of studies, insight was gained into the evidence itself, and how cultural knowledge featured among other types of knowledge generated in research on EoL care (for example relating to clinical practice or service development). This showed the areas of expertise in different countries and revealed gaps in knowledge.

## Results

### Literature Flow

**Figure 1:**
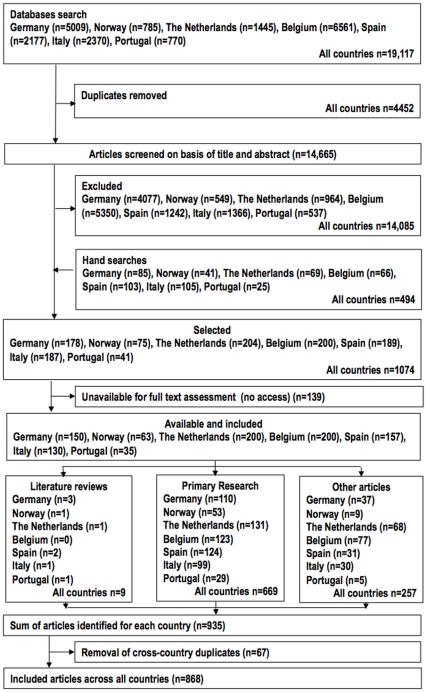
Flow chart of included countries.

See Figure 1 for the flow chart of included countries.

### Nature of the Evidence

A total of 868 papers were reviewed.

### Synthesis: the Netherlands

Identified papers were well distributed between 1992 and 2012. Articles published before 1992 were in Dutch and beyond the scope of this review. These were, however, well cited in the papers appearing in international journals. This already points towards the contested history of palliative care in the Netherlands: it is either seen as an underdeveloped area of expertise, when one considers its start with the establishment of hospices in the early 1990s [21], or as a field which already started to develop through work in nursing homes back in the 1960s [22].

The literature on the Netherlands provided information about a variety of settings in which EoL care is provided and the experience and role of health professionals involved [23], [24], [25], [26], [27], [28], [29], [30]. End of life care used to be part of the country’s highly developed home care system, with general practitioners playing an important role, which was further encouraged by national healthcare policy [25], [28]. Some studies described how the differences in EoL care settings affected the type of care provided [27], [29]. Despite the key role played by informal carers in home care, only two studies focused on their experiences [31], [32].

The majority of studies focused on medical EoL decisions (MELDs), reflecting the country’s unique situation, where euthanasia was legalised in 2002 (together with Belgium in 2002 and Luxemburg in 2008). Euthanasia and physician assisted suicide (PAS) were the most common topics of research in this area. A recent comparative study of the Netherlands and Belgium on the first five years of euthanasia legislation showed that there are differences in how the legislation is applied in each country [33]. Other MELDs such as non-treatment decisions [34] and palliative sedation [35], [36] were also explored. Considerable attention was devoted to the definitions, differences between, and incidences of, MELDS [37], [38], [39], [40]. A culture of tolerance towards euthanasia and PAS was described which had been embedded in Dutch society for over 100 years [41], [42].

Other articles explored the importance of self-governance for terminally ill people in a Dutch context [32]. Several aspects were explored regarding ethanasia, its emotional impact on physicians [43], patients’ reasons for requesting euthanasia [44], and the experiences of relatives and friends of patients who received euthanasia [45]. One study focused on the role of the euthanasia consultant who evaluates the criteria for careful practice and advises about palliative care [46]. An ethnography showed that a request for euthanasia often served a symbolic purpose and enabled open discussion about taboo subjects of death and suffering [47]. Similar findings came from subsequent studies [32], [48].

A recent study described the phenomenon of “self-directed deaths,” individually controlled methods to hasten death, and showed that frequencies were very close to physician assisted death in the same year [49].

Issues relating to communication revolved mainly around advance directives (ADs) [41], [50], [51], [52], [53], disclosure and information giving [26], [54], [55], [56], [57], [58], [59]. The issues relating to ADs were determined by the euthanasia situation. Public knowledge about ADs was found to be high. These are documents in which one can state the wish for euthanasia in certain cicumstances. [60]

Only three studies addressed EoL care relating to ethnic minority groups in the Netherlands. One study found that euthanasia was not less common, while symptom alleviation occurred less among this group [61]. Another study examined Dutch professional home care and the barriers to the use of this care for terminally ill Turks and Morrocans and their families [62]. It was shown that the latter group have conflicting ideas about ‘good care’ compared to their Dutch care providers and they found ‘palliative care’ a contradiction in terms due to their focus on cure. [63]

### Belgium

The majority (114) of articles were published between 2000 and 2010. The first publications appeared when the euthanasia debate had just started, resulting in the enactment of the euthanasia law in 2002. Unlike in the Netherlands, this debate was brief and palliative care played an important role in it. Although palliative care had only started five years before, it was very successful. The two movements developed in parallel. Shared workers were dedicated to both causes and this resulted in the model of ‘integral palliative care’ [64]. This model is inclusive of euthanasia, and as such it responds to the pluralist make up of Belgian society.

Against this background, research aimed at developing an understanding of place of care and death. Studies provided insight into different settings where people die in Belgium [65], [66], [67], [68], [69], the determinants of place of death [66], [69], [70], [71], [72], [73], [74], [75], and how place of death compared internationally [70], [74], [76]. One study focused on transitions between settings [77].

One key aspect of the health system in Belgium was that GPs tended to work alone and often had a long-standing and trustful relationship with patients. There was an emphasis on individualized care and the ability to choose one’s own doctors.

Place of death influenced medical EoL decisions (MELDs) [78].

MELDs was the most researched topic in the Belgian literature, mainly focusing on definition of concepts [79], [80], [81], [82], incidence of MELDs [69], [80], [83], [84], decision making [85], [86], including communication [87], key actors [88], [89], [90], and the role of health professionals [91], [92], [93], [94], [95], [96], [97], [98], [99], [100], [101].

These studies present evidence for or against the legal developments in Belgium. The studies that assessed MELDs in practice showed that the slippery slope effect had not materialized [65]. Also, with the availability of palliative care, euthanasia requests were less likely [102], [103], [104], [105], [106]. These requests were however not preventable in all cases. A recent study reported that 90% of physicians support euthanasia for terminal patients with extreme uncontrollable symptoms [107]. A study comparing practices of euthanasia and assisted suicide with the Netherlands, showed that there were significantly fewer cases in Belgium (1917) than in the Netherlands (10319), mostly in patients suffering from diseases of the nervous system, and in hospital [33].

Other topics related to MELDs included application of laws and other regulations, institutional written ethics policies, and opinions and attitudes [65], [83], [86], [101], [105], [108], [109], [110], [111], [112], [113], [114], [115], [116], [117], [118], [119], [120], [121], [122].

The articles on communication examined disclosure [123], [124], [125], [126], [127], [128], [129] communication with different actors [98], [127], [130], barriers to communication [124], [125], [131], [132] and ADs [96], [115], [116], [133], [134], [135], [136].

One paper looked at the approach to EoL care by different ethnic groups in Belgium and called attention to non-Western perspectives [137].

### Germany

The majority of articles were published within the last decade, with the remaining studies having been published between 1989 and 1999.

Home was identified as most people’s preferred place of death, even though the majority of deaths actually occurred in hospitals [138], [139], [140], [141]. The growing importance of nursing homes as a place of care and death, and the need to incorporate palliative care into these institutions, was highlighted [142], [143].

In cross-country comparisons German physicians were found to be more likely to exclude patients, patients’ families and non-medical staff from the decision making process [144], [145]. ADs was a well-covered theme. Studies explored awareness of ADs, use and compliance [138], [144], [145], [146], [147], [148], [149], [150], [151], [152] and desired level of bindingness [150], [153], [154], [155].

Medical end of life decisions was also a major theme in the literature. Active euthanasia is illegal in Germany and the National Board of Physicians rejects any liberalisation concerning active euthanasia [156]. PAS is not illegal in Germany. However, physicians have the responsibility to attempt to apply all medical measures to prevent death, making PAS unfeasible in practice [157]. The German Medical Association rejects PAS as against its ethos [157].

Cross-country comparisons found that Germans had relatively low acceptance of euthanasia given the secular and individualistic characteristics of the society [111]. This was ascribed to the use of the term ‘euthanasia’ by the Nazi regime [111]. Its association with Nazi medicine has been avoided by using different terminology. The preferred term ‘Sterbehilfe’(literally “help to die”), was found to be the same as that of active and passive euthanasia in the international literature [158], [159]

There was confusion among physicians and medical students regarding the difference between active and passive ‘sterbehilfe’, and the legality of assisted suicide [146], [160]. In contrast to the high levels of opposition encountered for PAS and euthanasia, there was a high level of acceptance of palliative/terminal sedation [157].

The German literature on EoL care reflects a lack of social consensus on all topics and by all stakeholders.

### Norway

One study was published in the early 1980s, ten were published in the 1990s and the vast majority (34) were published between 2000–2010. A topic frequently touched upon within the original studies was the proportion of deaths in institutional settings in Norway. Norway has the highest percentage of beds in nursing home facilities per capita in Europe, more than twice that of most European countries and the highest number of deaths in nursing homes and hospitals [161], [162]. Therefore the importance of palliative care provision within the nursing home setting was emphasised [163], [164]. In contrast to other international studies, EoL care in a Norwegian nursing home was perceived as professional and good by patients’ families [165]. Several studies also focused on different aspects of home care [162], [166], [167], [168], [169], [170], [171].

Studies of carers examined their characteristics [172], concerns [173], activities [174], effects of caring [162], [168], [171], [175], [176], and setting where the care was provided [168], [173].

One of the sub-themes relating to communication was the uncertainties experienced by doctors and nurses regarding disclosure [177], [178]_._ A general reluctance to talk about death was found [172], [179], and a 20 year study in one hospital showed that open discussion of death with patients had not increased over time [179]. This was ascribed to the Norwegian respect for privacy [172], although it could also be attributed to a strong death taboo.

Another communication issue concerns the stakeholders included in decision-making. Norwegian physicians have the ultimate responsibility for treatment decisions, and whether or not the views of other professionals were taken into account depended upon the individual physician’s views and the culture of the healthcare setting. Family members were often included in decision-making, which led to greater agreement concerning treatment options which was also the case for the understanding of the patient’s wishes.

Only one form of advance directive is available in Norway (the ‘Life Testament’), which has no legal status and is rarely used [172], [180]. Its incidence was studied with two studies on patients [172], [179] and two on health professionals [180], [181].

Treatment limitation was the most frequently studied topic amongst the original studies, including its incidence [179], [180], [182], [183], criteria for limiting life [172], [181], [184], [185], and ethical dilemmas surrounding decisions [180], [181], [182], [183], [185], [186], [187]. The role of nurses and physicians was examined and the effect of EoL decisions [185], [188], [189], [190].

Euthanasia and PAS are illegal in Norway and the practices are condemned in the Norwegian Medical Association’s ethical guidelines [186]. Norway’s Lutheran heritage, and a puritan ‘moral minority’, were said to influence the debate on euthanasia [191]. Norwegian physicians have more conservative attitudes than other Scandinavian and western countries in regard to treatment limitation, euthanasia and PAS [177], [180], [186]. The low level of ‘hastening death’ found in Norway was attributed to a cultural respect for the law, which prevented even physicians who held liberal ideas concerning euthanasia from carrying out euthanasia in practice [180]. In Norway palliative sedation and euthanasia were said to have only recently been differentiated, and guidelines for practice provided.

Frequent topics in the Norwegian literature were healthcare spending and accounting. Elderly people were repeatedly said not to receive a ‘just’ allocation of resources [172], [184], [189], [192]. Also, ethical dilemmas caused by the use of ‘high technology’ were frequently highlighted [188], [190], [192], [193].

### Spain

Only six of the 123 studies for Spain were conducted before 1990 and since then research on EoL care has increased. The main palliative care resource was home care teams, the second, in-patient units [14]. There were no general data on place of death: the identified studies presented a range of percentages of home deaths: from 22% for elderly people who died in Catalonia in 1998 to 50% for terminal cancer patients in Asturias in 1995 [194], [195], [196], [197], [198], [199], [200]. Percentages of healthy people who would like to die at home were generally higher than those who actually die at home [200], [201], [202], [203], [204], [205] nevertheless a recent survey showed that half of the population has preferences for specific care settings or hospitals for terminal patients [202]. There was greater consensus among healthcare professionals than among the general public that the home is the ideal place of death [206], [207], [208], [209].

More than 84% of the patients were cared for by family members, mainly daughters and wives [195], [198], [210], [211], [212]. A majority of the caregivers were found to be overloaded [212], [213] and did not have any economic help or enough information of the resources available [213], [214], [215]. Two concepts with negative connotations relating to the patient’s family were identified: the ‘conspiracy of silence’ [216], [217], [218], the partial or non-disclosure which is frequently attributed to family members; and, ‘claudicación familiar’ (family surrender), when patients die in hospital and not at home (the ideal place of death according to health professionals) [219].

Disclosure of information regarding diagnosis, prognosis and treatments was found to be the most frequently discussed in the literature and this was also the topic of the identified review [220]. International comparisons described southern European countries as partial and non-disclosure countries [221], [222], [223] and Spanish awareness studies suggested that this trend persisted over time [194], [204], [218], [224], [225], [226], [227]. On the other hand, studies with healthy populations show that preferences are evolving towards open disclosure [201], [202], [203], [204], [207], [228]. Intermediate positions were also found; the majority of doctors stated that they would inform the patient only in certain circumstances or if requested by the patient [206], [207], [208], [209], [228], [229], [230], [231], [232]. The two main obstacles to giving bad news were found to be acceptance of the wishes of the family, hence tolerating the ‘conspiracy of silence’ imposed by the relatives, and feeling uncomfortable to give bad news [217].

In Spain, the legal and administrative development of ADs is one of the most advanced in Europe [233]. Most doctors found the policies relating to their implementation a positive development [233], [234]. However the public’s knowledge and use of ADs was very limited [202], [235], [236], [237], [238].

In an international study Spain was shown to occupy an intermediate position in Europe regarding the acceptance of euthanasia among the general public [239], and the acceptance had risen since 1995 among the general public. [202], [240], [241]

In international comparisons, Spain was among the countries with the lowest prevalence of Do Not Resuscitate (DNR) orders, and among those where these were discussed less with the patient. There were low rates of treatment withdrawal [212], [242], [243], [244], [245]. In Spain the rates of all three practices were higher than in Portugal, Italy, Greece, and in the case of withdrawal of dialysis Spain was above Germany. National studies suggested even higher use of these MELDS [246], [247].

Terminal sedation was considered consistent with the traditional Spanish perception that unconsciousness is the ‘best way out’ [248], [249]. However, terminal sedation is still controversial in Spain. The legal proceedings against the Leganés Hospital Emergency Unit have demonstrated that the boundary between euthanasia and terminal sedation is not totally clear [250]. Part of the controversy concerns its use to manage existential and family distress, more common in Spain than in other countries [216].

Regarding feelings towards death and dying amongst both health professionals and the general public in Spain, fears related to pain were found to be the most important [203], [204], [251], [252], [253]. However morphine consumption per capita was below the European and global average [254]. Fears about death were found to be a major barrier to good EoL care [255].

Four research studies examined EoL experiences of migrants from Morocco [256], [257], Latinamerica [258] and UK [259] in Spain, and four overviews were found that covered EoL issues for migrants [260], [261], [262], [263].

### Italy

In Italy, there has been a steadily growing number of research studies since 1979. EoL care is delivered mainly by home care teams [264] and the number of hospices rose from four in 1996 [265] to 90 in 2005 [14] mainly due to new palliative care policies since 1999 [266]. From the different factors related to place of death within Italy [70], [267], [268], region was found to be the most determining: in the south of Italy, the percentage of home deaths was 94% for cancer patients [267].

As in Spain, more than 85% of cancer patients’ caregivers were relatives [269], [270], [271], [272], and caring had an important impact in their quality of life [270], [271], [272], [273], [274]. Nevertheless they were frequently characterised in a negative way as barriers to full disclosure and limitation of non-useful treatment [275], [276], [277].

Also as in Spain, most studies from Italy focused on disclosure of information, with a review from 2004 on this topic [278]. Awareness studies published between 1994 and 2009 showed that a trend of partial and non disclosure persisted [279], [280], [281], [282], [283], [284], [285], [286]. The choice of partial or non-disclosure arises within families, independently of patients’ requests [287]. Other sources however suggest that physicians preferences are moving towards full disclosure [277], [288], [289], [290].

ADs are not legally recognised. Recently the parliament approved a non-binding law that the patient does not express his/her ‘will’, but ‘wish’, and this was after there had been intense debate, influenced by public opinion, concerning a number of high-profile cases [291], [292], [293], [294].

Europe-wide surveys of the general public found that Italy was among the countries with the lowest acceptance of euthanasia [112], but the differences between Catholic believers and non-believers were higher than in other European countries. Death was less likely to be preceeded by a MELD than in other European countries [295] whereas terminal sedation was more frequent. A recent paper showed that there is still low and often incorrect awareness of palliative care among the general public [296].

Many of the studies focused on pain management and showed low opioid consumption [254] and a significant proportion of patients not receiving appropriate treatment [297], [298], [299], [300], [301], [302]. However knowledge about pain and analgesics was found to have improved [303], [304], [305].

Four articles focused on Italians as minorities in other countries [306], [307], [308], [309]. Only one original study gave specific information on immigrants [310] and one overview presented the islamic perspective in pediatric biomedical ethics including EoL [311].

### Portugal

More than half of the articles were published in the last five years (11), with the remaining studies having been published between 1987 and 2003. The development of services and research started relatively late in Portugal where the first palliative care unit only opened at the end of 1994 [312]. There were country-wide statistical data on place of death: with almost one-third of all deaths occurring at home [313].

As in Spain and Italy, caregivers needs included information, time to relax and economical support and care [314]. Following again the southern Europe trend, disclosure was one of the main themes explored in original studies. Two studies, both conducted in Porto, described greater patient awareness (60–69%) and desire for information than in Spain or Italy [315], [316].

Portugal, like Italy, is among the countries with the lowest public acceptance of euthanasia [112]. A study from 2009 reported that up to 39% of oncologists favoured the legalisation of euthanasia [317]. The use of terminal sedation was lower than in other countries. Delirium was the most common grounds for initiating sedation while pain was an uncommon reason [315]. Portugal’s opium consumption was found to be above the European and global average [254].

A recent publication calls for attention to informal caregiving for older people [318]. Another recent study focuses on palliative care physicians’ views of ADs, and found them relevant to ethical decision making [319].

## Discussion

### Socio-Cultural Issues in EoL Care: What the Evidence Says

There is still little agreement about what constitutes EoL care in Europe. Researchers, practitioners and policy makers have different understandings of its scope, definitions, goals and approaches [320], [321] and there are limited resources for its development. Identifying and analysing diversity in understandings and practices in EoL care in the different countries is essential for reaching consensus on EoL care, and for achieving workable standards.

In this scoping exercise we found a diverse body of evidence on socio-cultural issues in EoL care with differences in meanings and priorities in each of the countries (see Table 3 and Table S1).

**Table 3:** Numbers and percentage of studies identified per theme across studies.

Country	Type of article		Total	Setting	Caregivers	Communication	Medical EoL Decisions	Minority Ethnic Groups*	Knowledge, Attitudes and Values
***Germany***	***Reviews***	**3**	N	0	0	0	2	0	2
			%	**0**	**0**	**0**	**67**	**0**	**67**
	***Original studies***	**110**	N	18	6	43	49	2	36
			%	**16**	**6**	**39**	**45**	**2**	**33**
	***Overviews etc.***	**37**	N	2	3	11	22	2	12
			%	**5**	**8**	**30**	**59**	**5**	**32**
***Norway***	***Reviews***	**1**	N	0	0	0	1	0	1
			%	**0**	**0**	**0**	**100**	**0**	**100**
	***Original studies***	**53**	N	22	12	21	30	0	31
			%	**42**	**23**	**40**	**58**	**0**	**60**
	***Overviews etc.***	**9**	N	3	0	2	6	0	2
			%	**33**	**0**	**22**	**67**	**0**	**22**
***Belgium***	***Reviews***	**0**	N	0	0	0	0	0	0
			%	**N/A**	**N/A**	**N/A**	**N/A**	**N/A**	**N/A**
	***Original studies***	**123**	N	25	3	14	61	11	10
			%	**20**	**3**	**11**	**50**	**9**	**9**
	***Overviews etc.***	**77**	N	0	1	6	50	4	13
			%	**0**	**1**	**8**	**65**	**5**	**17**
***Netherlands***	***Reviews***	**1**	N	0	0	0	1	0	0
			%	**0**	**0**	**0**	**100**	**0**	**0**
	***Original studies***	**131**	N	16	9	24	70	3	35
			%	**13**	**7**	**18**	**54**	**2**	**27**
	***Overviews etc.***	**68**	N	0	1	4	59	0	24
			%	**0**	**1**	**6**	**87**	**0**	**35**
***Spain***	***Reviews***	**2**	N	0	0	1	0	0	1
			%	**0**	**0**	**50**	**0**	**0**	**50**
	***Original studies***	**124**	N	33	18	38	29	3	33
			%	**27**	**15**	**31**	**24**	**2**	**27**
	***Overviews etc.***	**31**	N	0	0	5	5	2	25
			%	**0**	**0**	**16**	**16**	**6**	**81**
***Italy***	***Reviews***	**1**	N	0	0	1	0	0	0
			%	**0**	**0**	**100**	**0**	**0**	**0**
	***Original studies***	**99**	N	19	11	38	24	3	25
			%	**20**	**11**	**38**	**25**	**3**	**25**
	***Overviews etc.***	**30**	N	1	1	21	7	2	3
			%	**3**	**3**	**70**	**23**	**7**	**10**
***Portugal***	***Reviews***	**1**	N	0	0	1	0	0	0
			%	**0**	**0**	**100**	**0**	**0**	**0**
	***Original studies***	**29**	N	0	6	11	9	0	5
			%	**0**	**21**	**38**	**33**	**0**	**19**
	***Overviews etc.***	**5**	N	0	0	1	2	0	1
			%	**0**	**0**	**20**	**40**	**0**	**20**

This reflects a situation where EoL care has developed in different directions since the unique ideas of the hospice movement found resonance in Europe. The initial concept of palliative care has changed through its increasing contact with mainstream medicine in the different countries [322] and with the cultural traditions relating to health, illness, death, dying, bereavement, and ideas about care, the family, and the duties of medicine and society.

This scoping exercise revealed practices in EoL care that attest to cultural differences in ideas of best practices in EoL care. Disclosure practices in Mediterranean countries contradict the obligation to open information about diagnosis and are influenced by the continuous presence of the family in EoL care [323]. This can cause conflicts between the norms prevailing in the medical profession on the one hand and physicians’ and families actual practices on the other [221], [222], [223]. Also, the focus on cultural identity may be due to self-reflection as a consequence of the process in which palliative care is incorporated into national health systems. Aspects of the hospice movement’s particular philosophy can be experienced as foreign, for example the emphasis on awareness as part of good death contradicts the traditional Spanish ideas about dying well [248]. In other instances its strong moral values have come into contact with alternative conceptions of good care, which were based on professional experience of care for the dying developed over long periods of time. Here we think of the Dutch situation where euthanasia has developed as acceptable as a last resort and has long since been a topic that can be discussed openly [324]. The research generated in these seven countries on cultural issues at the EoL is directed towards the countries autochthonous cultural traditions and practices.

Although this scoping exercise approached the evidence by country, this does not mean we interpret ‘culture’ exclusively in terms of ‘national culture’ where cultural differences are aligned with the territorial borders of the nation state. However, for the scoping of this field we think this approach was justified. First, the scoping exercise was exploratory; no previous work has attempted to map cultural issues in EoL care across different European countries. Second, because we understand culture as an abstract notion, rather than a concrete set of values, beliefs, attitudes, opinions or other ethnic features we did not determine a priori any factors that could constitute culture. Third, regarding the topic of EoL care, we could follow national boundaries as there are distinct approaches between countries which are the result of different institutional forms of health care and a variety of ways of organising the professions that are in charge of care provision at the EoL. The reviews confirm that there are clearly distinguishable national cultures of EoL care.

This scoping study situated the identified literature in time, providing insight into a country’s research activity vis-à-vis its development of services. This shows (with the exception of the Netherlands, which already had a research tradition in EoL care) the first studies appearing not much later than the establishment of services on the European continent, with a growing production towards more recent times.

### Contrast with the UK

The findings from this review on culture and EoL care from the seven European countries contrast with the UK research activity in this area, where EoL care has developed considerable expertise on ethnic minority groups. Recently a review of original studies and a review of reviews on this topic was published (also in the context of this project), which represents a body of research that was produced in response to the recognition of inequities in access to healthcare and the quality of services provided related to patients’ ethnicity [18], [19]. Thirteen reviews were identified in this area of which four reviews were commissioned to directly influence policy and this already shows the recent interest in these issues in the UK [18]. The 45 original studies focused for a great part on the need to develop ‘cultural competence’ in health care [19].

In contrast, very little attention has been paid to cultural issues of ethnic minorities in EoL care in the European countries included in this review. In the Netherlands we found that some pioneering work had started in this area, and in the other countries there were a few scattered exceptions.

This scoping of the literature informs about the gaps in the evidence on culture and EoL care and this points to future needs for research for the further development of the evidence-base. In the UK, apart from the research on ethnic minority groups, a vast literature exists on EoL care and this has not yet been reviewed with attention to socio-cultural issues. This was also not possible in this project due to time and resource constraints. Such a study could shed light on the culture-specific pre-occupations with EoL care in the UK. It could show the dominant concerns towards care at the end of life and the configurations of positions towards these concerns in a diversity of contexts and settings. It is important to attend to culture and its uses in a broad sense so that it includes the majority culture as this will reveal that particular well-established practices are in fact culturally and historically situated. When these practices are then compared with those of other countries (as we did in this scoping exercise) it can show why certain practices become normalised while others remain unrecognised or become contested. Insights such as these lead to awareness of cultural differences and can enhance international collaborations.

### The Nature and Quality of Research in EoL Care and the Significance of Cultural Knowledge

The insights from this scoping exercise contribute to the debate about the quality and nature of research in palliative care. As a consequence of the evidence-based medicine movement, biomedical research has been favoured in palliative care. This is reflected in what is considered high quality research where criteria of strength are used according to the potential for eliminating bias [325]. Recently, these classification levels of evidence have been debated in areas of health research where these criteria are not representative of quality [326]. People at the end of their lives need care which is holistic and individual to address the patient and family’s complex problems, and these can not be grasped by methodological approaches that exclude contextual factors. The challenges presented by people at the end of their lives to enrolment and participation in trials in terms of retainment and ethical considerations lead to the exclusion of the most vulnerable, which is the group that is of most relevance to palliative care. Clinical practice guidelines based on the effectiveness results of RCTs have the danger of increasing existing health inequities [327]. Socio-cultural knowledge is important in EoL care and we need research that generates understanding of the ways these affect illness experiences and caring and that enables the building of a discipline that is capable to respond to the needs of diverse and changing communities.

### Limitations

The literature from Norway was limited to publications in English. No electronic searches were carried out with other than English terms in the main databases. However, publications identified from Spain, Italy, Portugal, Belgium, the Netherlands and Germany in their respective national languages from the vernacular databases searched or those obtained by hand searches were included in the review.

### Recommendations

On the basis of the findings of this literature scoping on cultural issues in EoL care in seven European countries we make the following recommendations for future research:

- The mapping and investigation of the literature has generated insight into cultural differences in understanding, priorities and expertise relating to EoL care across Europe. The analysis of the number and the type of studies (see Figure 1 and Table 3) serve as a systematic basis for further more focused analysis.
- The gaps identified in the evidence point to areas that should be the focus of exploratory studies, and the better-represented themes (summarized in Table S1) can inform the research questions of other systematic reviews, or particular topics can be further interrogated or complemented with new studies.
- Given the current expansion of EoL care into new areas, this field of research should be given due attention beyond the countries included in this review, and beyond Europe, on a global level.
- Research on cultural issues in EoL care needs to start from a well-informed understanding of the notion of culture to avoid stereotyping, which was a consequence of some previous research.
- The lack of research on ethnic minorities’ views, experiences, and practices is striking and deserves future study.
- Dominant cultural ideas equally need to be subjected to cultural investigation, which will uncover ideological interests and the way that some taken-for-granted practices are the product of wider forces.
- Robust multi-country studies in this review confirmed the existence of major cultural differences but sometimes did not explain the reasons for these differences. It is therefore advisable to explore these cultural issues more deeply through in-depth qualitative or mixed-methods studies.
- Future empirical evidence in this field is needed to serve as a basis from which to develop a more robust understanding of theoretical concepts related to culture (for example cultural competence) and EoL care (for example suffering, the experience of symptoms, and dignity).

### Conclusions

This scoping of the literature is a first comparative exploration of the cultural differences that exist in the understanding of EoL care in these countries. There was very little work in the evidence we unearthed specifically looking at cultural issues. With the exception of some pioneering work on EoL care for ethnic minority groups in a few countries, no expertise had developed in this area. European countries wrote about their ‘own’ cultural traditions and practices.

This scoping also critically appraised the research evidence on cultural issues in EoL care produced in the different countries to throw light on its adequacy as a basis for the further development of EoL care. The work on culture presented here provides an understanding of the evolution of the concept of palliative care across several European countries, shows the different cultural norms that influence care at the EoL and gives a view of the existing diversity in what is considered good care. This type of knowledge is a legitimate and necessary part of what constitutes best evidence for the improvement of EoL care in the future.

## Supporting Information

Table S1 **Sub-themes of culture and EoL care across countries.**
(DOC)
