# Supplementary material for: Culture and End of Life Care: A Scoping Exercise in Seven European Countries
Source: PLoS One. 2012 Apr 3;7(4):e34188. doi: 10.1371/journal.pone.0034188 (PMC3317929; doi:10.1371/journal.pone.0034188)
Supplement: Table S1 — Sub-themes of culture and EoL care across countries. (DOC) [file pone.0034188.s001.doc]

Table S1: Sub-themes of culture and EoL care across countries

|  | UK | Germany | Norway | The Netherlands | Belgium | Spain | Italy | Portugal |
| --- | --- | --- | --- | --- | --- | --- | --- | --- |
| Setting | Inequalities in service use for minority ethnic groups (MEG) | Discrepancy between wish for death at home and actual place of death in hospital.  Low awareness of hospice care.  Need to develop EoL care in nursing homes. | Place of care and death in institutions with low provision of palliative care. | Policy encourages the provision of EoL care at home which is highly developed and GPs fulfill an important role. GPs trained and supported by specialized palliative care consultants. | Place of death and its determinants is well documented and researched, also put in comparative perspective with other countries. | Home care as the main resource.  Palliative care units preferred above hospices. | Home care as the main resource.  Number of hospices increased significantly in the last decade. | Palliative care developed only in the 90s and is mostly organized in acute hospitals. |
| Caregivers | Support needs for MEG carers | Very little studies found. | Very little studies found. | Very little studies found. | Family involvement in decision making at the EoL. | Strong family involvement in EoL care. Concepts of ‘conspiracy of silence’, referring to non-, or partial disclosure.and ‘claudicacion familiar’ (family surrender) referring to patients dying at hospital due to families ‘surrendering’ at the EoL. | Strong family involvement in EoL care. Concept of ‘conspiracy of silence’ referring to non-, or partial disclosure. | Caregiving by female relatives and its burden. |
| Communication | Different disclosure norms for MEG  Lack of info on services in different languages | Patients’ wish for open communication about diagnosis and prognosis.  Professionals’ difficulties to communicate about the end of life.  Knowledge, use of and compliance with advance directives | Problems in patient-physician communication. Reluctance to talk about death | Issues regarding ADs were determined by the euthanasia situation. | Disclosure, communication with different actors, barriers to communication and ADs.  ADs have legal standing but there is a need for a clearer protocol. | Non- and partial disclosure.  ADs well developed administratively. | Non- and partial disclosure. Non binding ADs | In big cities, greater desire for information than other Mediterranean countries. |
| MELDs |  | Diversity of opinions regarding euthanasia and PAS.  Acceptance of palliative care and terminal sedation.  Position towards EoL decisions is determined by the situation in the neighbouring low countries and the history of the holocaust. | More professionals’ conservative attitudes towards euthanasia and death than other European countries.  Euthanasia and PAS were only recently differentiated. Clarity on palliative sedation needed.  Ethical issues caused by palliative sedation. | Definition and mutual differentiation, and incidences of MELDS.  Recent  phenomenon of self-directed dying as an organized movement. | Definition of concepts, incidences, decision making.  Applications of laws and other regulations after the legalization of euthanasia. Instutional ethics policies. | Public acceptance of euthanasia increased since 1995, reaching 81% in 2009.  Debate about palliative sedation. Also accepted to manage existential and family distress. | Lowest acceptance of euthanasia Europe-wide.  Public acceptance polarized between believers and non believers.  Low number of patients dying after a MELD except for palliative sedation. | Lowest acceptance of euthanasia Europe-wide. |
| Minority ethnic groups | Development of notion of cultural competence.  Training needs for professionals recognised | No studies found | No studies found | Recently 3 studies on EoL practices and experiences with care of non-western groups appeared. | Conference abstracts documented EoL care practices by different ethnic groups. | 4 studies in the last 10 years, outlining the new challenges | Very scarce literature, possibly due to the recent influx of immigrants | No studies found. Possibly due to recent development of EoL care,. |
| Know  ledge, atti  tudes, | Differences in the interpretation of symptoms and their management between professionals and MEG.  Stigma | Need for further training in palliative care. Need for provision of psycho-social care.  Lack of social consensus on all topics. | Mutual trust of patients and families in health professionals and vice versa, and towards society result from Lutherian and communitarian ideals.  The elderly and dying failed to receive a just allocation of resources due to increased consumerism and professionals prioritizing the most urgent medical issues. | Contested history of palliative care, either a disregarded area of expertise, when one considers its start with the establishment of hospices in the early 1990s, or as a field which started to develop through work in nursing homes in the sixties.  Importance of patient autonomy. | Palliative care framework embraces the option for euthanasia.  Both concepts –palliative care and euthanasia-developed in synergy. | Catholicism influenced attitudes towards life and death. Secularisation of society was documented.  Low opioid consumption. | Catholicism influenced attitudes towards life and death.  Secularisation of society was documented.  Significant proportion of patients not receiving appropriate pain control . | Portugal above European average of morphine consumption. |
